# Asymmetric Unilateral Vestibular Perception in Adolescents With Idiopathic Scoliosis

**DOI:** 10.3389/fneur.2019.01270

**Published:** 2019-12-03

**Authors:** Emma J. Woo, Gunter P. Siegmund, Christopher W. Reilly, Jean-Sébastien Blouin

**Affiliations:** ^1^School of Kinesiology, University of British Columbia, Vancouver, BC, Canada; ^2^MEA Forensic Engineers & Scientists, Richmond, BC, Canada; ^3^British Columbia Children's Hospital, Vancouver, BC, Canada; ^4^Faculty of Medicine, University of British Columbia, Vancouver, BC, Canada; ^5^Djavad Mowafaghian Centre for Brain Health, University of British Columbia, Vancouver, BC, Canada; ^6^Institute for Computing, Information, and Cognitive System, University of British Columbia, Vancouver, BC, Canada

**Keywords:** adolescent idiopathic scoliosis, vestibular function, asymmetry, etiology, electrical vestibular stimulation

## Abstract

The cause of Adolescent Idiopathic Scoliosis (AIS) remains unclear, but one proposed cause of AIS is asymmetric vestibular function and the related descending drive to the spine musculature. The objective of this study was to determine if asymmetric vestibular function is present in individuals with AIS. Ten individuals with AIS (8F, 2M) and 10 healthy age- and sex-matched controls were exposed to 10s-long virtual rotations induced by monaural or binaural electrical vestibular stimulation (EVS), and 10s-long real rotations delivered by a rotating chair. Using a forced-choice paradigm, participants indicated their perceived rotation direction (right or left) to stimuli of varying intensity. A Bayesian adaptive algorithm adjusted the stimulus intensity and direction to identify a stimulus level, which we called the direction recognition threshold, at which participants correctly identified the rotation direction 69% of the time. For unilateral vestibular stimuli (monaural EVS), the direction recognition thresholds were more asymmetric in all participants with AIS compared to control participants [(0.22–1.00 mA) vs. (0.01–0.21 mA); *p* < 0.001]. For bilateral vestibular stimuli, however, the direction recognition thresholds did not differ between groups for either the real or virtual rotations (multiple *p* > 0.05). Previous reports of semicircular canal orientation asymmetry in individuals with AIS could not explain the magnitude of the vestibular function asymmetry we observed, suggesting a functional cause to the observed vestibular asymmetry. Thus, the present results suggest that a unilateral vestibular dysfunction is linked to AIS, potentially revealing a new path for the screening and monitoring of scoliosis in adolescents.

## Introduction

Adolescent idiopathic scoliosis (AIS) is the most prevalent subtype of idiopathic scoliosis, with the onset of scoliotic curvature occurring between 10 and 16 years old (1). Although the exact cause of AIS remains unknown, one possible cause is sensorimotor dysfunction (2, 3). More specifically, an asymmetry in vestibular function could generate imbalanced descending drive to the spinal musculature and thus contribute to the development of scoliosis (4–7). Anatomical asymmetries in the semicircular canal geometry are present in AIS patients (8, 9) and corresponding functional semicircular-canal asymmetries in response to caloric vestibular stimuli have been observed by some researchers (6, 7). Other researchers, however, have failed to identify significant differences in canal function from controls (9, 10), leaving uncertain the contribution of vestibular asymmetry to AIS.

**Figure 1:**
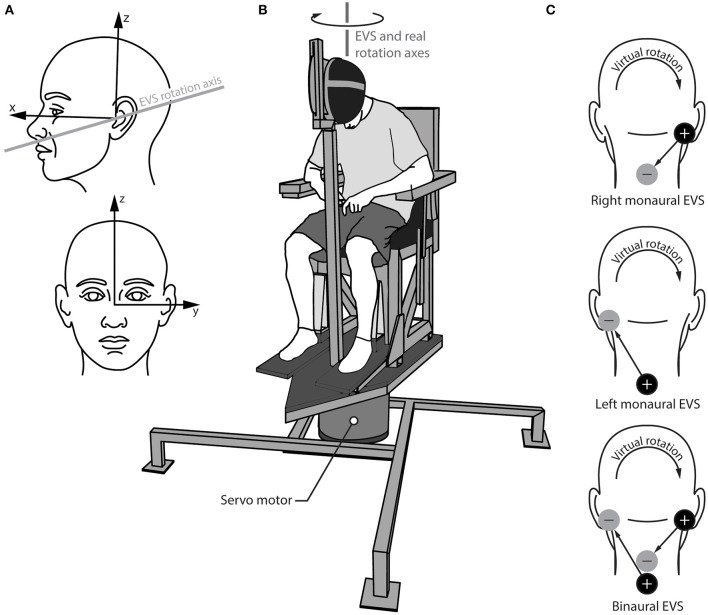
Experimental set up the for real and virtual rotations. **(A)** Head reference frame showing the orientation of the virtual axis of rotation for electrical vestibular stimulation (EVS); **(B)** rotary chair and helmet set up showing the virtual EVS rotation axis and the real rotation axis aligned vertically in the lab frame; **(C)** electrode configurations for the right and left monaural stimulations (top two schematics) and the binaural stimulation (bottom schematic). The cathode (+) and anode (–) locations depict the configuration for the positive waveform, which induces the virtual rotation directions shown. The arrows from the cathode to the anode show the direction of the positive current.

Caloric vestibular stimuli can assess unilateral semicircular canal function, but it mainly targets the horizontal semicircular canal (11–15). Another method to assess asymmetric vestibular function is electrical vestibular stimulation (EVS) applied over the mastoid processes (16–18). EVS activates the primary vestibular afferents from all semi-circular canals and otoliths through direct activation of the afferents or hair cells (19–21), with irregular afferents showing a larger sensitivity to the electrical stimuli (22–24). EVS is most often applied using two electrodes: a cathode (+) electrode placed on one mastoid process and an anode (–) electrode placed on the other mastoid process. Afferents under the cathode electrode increase their firing rate and afferents under the anode electrode decrease their firing rate (23, 25). As a result of these firing rate changes, EVS induces a sensation of head motion even though no actual head motion occurs. When EVS is applied bilaterally over the mastoid processes (i.e., in a binaural bipolar EVS configuration), the net virtual rotation it induces is about a vector pointing posteriorly and ~17–19° above Reid's plane with negligible net linear acceleration from the otolith signals (17, 18, 26) (Figure 1A). This virtual motion is similar to head roll in head-centered coordinates. When participants maintain a neutral head posture (i.e., head upright), EVS generates the illusion of head roll without the corresponding change in the gravitational signal from the otoliths. This mismatch is foreign to the brain, and generates a concurrent illusion of an interaural linear acceleration of the head (27) due to the brain's internal representation of gravity (28–30). However, when seated participants flex their head forward to orient the EVS vector vertically (i.e., parallel to the direction of the Earth's gravity), no change in the gravitational signal is expected by the EVS-induced rotation, and thus binaural bipolar EVS induces a perception of whole-body yaw rotation that is indistinguishable from a real yaw rotation in the same head-flexed posture (18, 31) (Figure 1B).

Other electrode configurations have also been used with EVS. When the electrodes are instead applied to one mastoid process and the C7-T1 spinous processes (i.e., in a monaural EVS configuration), EVS is thought to preferentially activate the primary vestibular afferents ipsilateral to the stimulated mastoid process (32). As before, the firing rate of all ipsilateral vestibular afferents increases if the cathode is applied to the mastoid process and decreases if the anode is applied to the mastoid process. This ability to preferentially stimulate the vestibular organs unilaterally provides an avenue to quantify vestibular asymmetry. Here, we combine monaural EVS with direction recognition thresholds (16, 33, 34) to quantify vestibular asymmetry and its association with AIS.

The aim of this study was to investigate vestibular asymmetry in individuals with AIS and compare this asymmetry to age- and sex-matched control participants. Direction recognition thresholds for virtual rotations using EVS and real rotations while seated in a chair were quantified using a forced-choice recognition task (16). Asymmetric vestibular function was estimated by computing the absolute difference of the recognition thresholds between the left and right monaural EVS configurations. We hypothesized that participants with AIS would exhibit an asymmetric vestibular response to monaural electrical vestibular stimuli compared to control participants, who would not exhibit such asymmetry.

## Methods

### Subjects

Ten otherwise healthy subjects with AIS and 10 healthy age- and sex-matched controls with no ontological or neurological disorders participated in this study (Table 1). Written and informed assent and consent were obtained from the participants and their legal guardians, respectively. All procedures conformed to the Declaration of Helsinki and were approved by the Clinical Research Ethics Board of the University of British Columbia (H16-00801; date of approval: 6/7/2016).

**Table 1:** Experimental group characteristic comparison.

	**Participants with AIS mean (SD)**	**Control participants mean (SD)**	***p*-value**
Sex	8F, 2M	8F, 2M	–
Age (year)	14.1 (1.5)	14.1 (1.7)	1.00
Height (cm)	162.1 (6.2)	163.0 (9.1)	0.79
Weight (kg)	52.1 (8.6)	49.1 (7.0)	0.39

Cobb angles for the AIS subjects were measured from an antero-posterior radiograph taken within 3 months of the study. The Cobb angle is the angle between the superior endplate of the most rostral vertebra and the inferior endplate of the most caudal vertebra of the largest lateral deviation in the thoraco-lumbar spine (see inset in **Figure 5**). For the two AIS subjects tested post-surgery, pre-surgery Cobb angles were measured 3 and 7 months prior to testing (Table 2). Control participants were screened for scoliosis using the Adams Forward Bend Test (35) and their trunk rotation angle was measured using a smartphone application (Scoliometer, Health in Your Hands, Singapore; iPhone 6S, Apple Inc., Cupertino, CA, USA) at three locations: upper thoracic (T3/T4 vertebrae); main thoracic (T6-T9 vertebrae); and thoracolumbar (T12/L1 vertebrae) (36, 37). Control participants with a trunk rotation angle above 5° were excluded.

**Table 2:** AIS participant's descriptive characteristics of major curvature.

**Sex**	**Surgery prior to testing**	**Cobb angle**	**Location**	**Direction^**†**^**
F	No	26°	T5-T11	Right
F	No	29°	T5-T11	Right
F	No	32°	T5-T11	Right
F	No	34°	T5-T11	Right
F	No	35°	T5-T11	Right
F	No	37°	T8-L2^*^	Left
F	No	42°	T10-L3^*^	Left
F	Yes	61°^**^	T9-L3^*^	Left
M	No	66°	T6-T12	Right
M	Yes	79°^**^	T5-T12	Right

*M, male; F, female*.

† *Direction of deviation from the mid-sagittal plane of the major convex curvature*.

* *Thoracolumbar curvature*.

** *Pre-operative curvature measurement*.

### Procedures

All subjects performed four test series: one involving real rotations (mechanical stimulus) and three involving virtual rotations (EVS). For all test series, subjects were seated in a chair and wore a helmet that was clamped to the seat frame with their head flexed forward ~71–73° to orient their EVS vector vertically [Figure 1B; (17)]. This head orientation does not change the perception sensitivity to real rotations (16) and avoids the perception of EVS-induced linear accelerations because the EVS rotation vector is parallel to gravity (27). Subjects wore a blindfold, earplugs, noise-canceling headphones (Quiet Comfort 25, Bose, Framingham, MA, USA) and had memory foam padding placed between their trunk and the seat frame to minimize other somatosensory cues.

**Figure 2:**
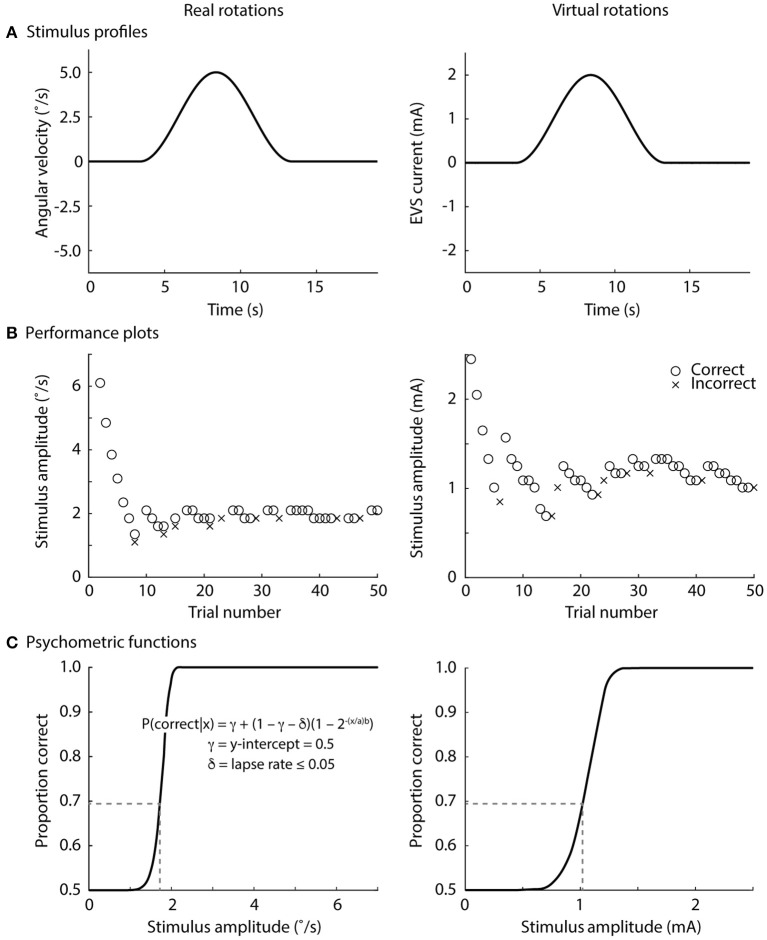
Direction recognition thresholds for real and virtual rotations. **(A)** Stimulus profile, single cycle raised cosine curve at 0.1 Hz; **(B)** exemplar performance plots, correct responses denoted by o's and incorrect responses denoted in x's; **(C)** exemplar psychometric functions fitted to the data in the exemplar performance plots. See text for an explanation of the psychometric equation. The stimulus level at 0.69 on the proportion-correct axis is the direction recognition threshold (dashed line). The lapse rate was ≤0.05 for the online analysis (during the experiments), but was set to zero for post-processing.

Real rotations were delivered using a custom-built rotary chair (Figure 1B) driven by a motion controller (PXI-7350 and UMI-7774, National Instruments, Austin, TX, USA), servo amplifier (SGDV-200A01A, Yaskawa, Japan) and servo-controlled AC motor (SGM7D-2ZN, Yaskawa, Japan; angular resolution 0.00034°). We generated single cycle raised-cosine angular velocity signals (*f* = 0.1 Hz) ranging between 0.1 and 15°/s with 0.25°/s increments for the Bayesian adaptive procedure (see Direction recognition threshold estimation) implemented in LabVIEW using the NI motion programming suite (v2013, National Instruments, Austin, TX, USA) (Figure 2A).

Virtual rotations were delivered using EVS in two monaural configurations (left and right) for preferential unilateral activation of the vestibular afferents and one binaural configuration for bilateral activation of the vestibular afferents (Figure 1C). For the binaural configuration, the two monaural electrode pairs were stimulated simultaneously. This approach ensured similar current paths for the monaural and binaural electrode configurations (38). Carbon rubber electrodes (9 cm^2^) were placed over the mastoid processes and the C7/T1 vertebrae and were secured with surgical tape. To minimize non-vestibular cues associated with the electrical stimuli, the skin under the electrodes was anesthetized with AMETOP (tetracaine HCl gel 4%, Smith & Nephew Medical Ltd., Hull, UK) applied about 30 min prior to electrode placement. Prior to placement, all electrodes and electrode locations were cleaned with alcohol and the electrodes were coated with 1 cm^3^ of conductive electrode gel (Spectra 360, Parker Laboratories, Inc., Fairfield, NJ, USA) to maintain similar electrode impedance (~5 kΩ; F-EZM5, Grass Instruments, West Warwick, RI, USA). The electrical stimuli consisted of single cycle raised-cosine EVS signals (*f* = 0.1 Hz; Figure 2A) ranging between 0.05 and 5 mA with 0.08 mA increments for the Bayesian adaptive procedure (see Direction recognition threshold estimation) implemented in LabVIEW. The signals were sent from a data acquisition board (PXI-6289, National Instruments, Austin, TX, USA) directly to either one (monaural) or both (binaural) constant-current isolation units (STMISOL, Biopac System, Goleta, CA, USA) that were connected to the electrodes. We measured the current and voltage applied to the participants (STM100C and STM100V, Biopac Systems, Goleta, CA, USA) to ensure that the electrode impedance was similar for the left and right monaural configurations as well as between subject groups.

### Direction Recognition Threshold Estimation

Participants completed the real rotation condition first in order for the AMETOP cream to anesthetize the skin. The order of the subsequent three virtual rotation conditions was then randomized. Before each of the real and virtual rotation conditions, participants received five practice trials at 5°/s (real rotations) and 2 mA (virtual rotations). All participants correctly perceived the direction of rotation in all practice trials. For each condition, subjects received a series of 50 stimuli (total 200 stimuli) and were told when a stimulus would be delivered. After each stimulus, participants were asked which direction they rotated (left or right; forced-choice task) and then told if their response was correct. We provided this feedback to maintain the participants' engagement in the task. Rotation directions were randomized across trials. The directions of the virtual rotations were defined as a right rotation for the cathode and anode configurations shown in the Figure 2, and a left rotation for the reversed polarity.

To find each subject's direction recognition threshold (DRT), the peak amplitude of the stimuli was varied across trials using a Bayesian adaptive procedure (16, 39). All participants experienced 7.35 °/s or 2.5 mA for their first real and virtual rotation, respectively; most subsequent stimuli determined by the adaptive Bayesian procedure were below these values because participants provided correct responses at these initial stimulus levels (see exemplar data in Figure 2). From each participant's performance, a psychometric function relating the peak amplitude of the vestibular stimulus (in °/s or mA) to the proportion of correct direction recognitions was fitted (Figures 2B,C). A sigmoidal psychometric function for each participant was parameterized as a modified Weibull function (40) (see equation in Figure 2C). An intercept (γ = 0.5) was chosen because guessing would result in 50% correct responses in a forced-choice task. For the online adaptive procedure, we allowed a range of lapse rates (δ ≤ 0.05) to account for the realistic possibility of occasional attention lapses (16). The inclusion of this lapse rate minimized the risk that the online adaptive procedure would deliver suboptimal stimuli if a subject incorrectly responded to a large supra-threshold stimulus. Since lapse rate was not a parameter of interest, we marginalized lapse rate over all of its possible values during the subsequent off-line analyses, effectively assuming a 0% lapse rate (δ = 0) for generating the final psychometric function. The parameters a and b were solved to best fit each subject's data, and the recognition threshold was then defined as the stimulus level (real rotations: °/s; virtual EVS rotations: mA) at which the subject correctly discriminated rotation direction with a 69% probability. This probability level corresponds to a discriminability index equal to 1 for a one-interval direction recognition task (41). Here, discriminability index refers to the theoretical separation between response distributions for right and left rotations, normalized by the standard deviation which was assumed to be equal for both directions (16).

### Statistical Analysis

To test our hypothesis, we first compared the absolute value of subject-by-subject differences between the left and right monaural EVS direction recognition thresholds (i.e., |DRTleft -DRTright|) to assess any asymmetry in unilateral vestibular perception between the AIS and control groups. Then, we compared the lower detection recognition thresholds [i.e., min(DRTleft, DRTright)] between the AIS and control groups as well as the higher detection recognition thresholds [i.e., max(DRTleft, DRTright)] between the AIS and control groups. These secondary comparisons were performed to assess whether any differences in vestibular asymmetry were the results of absolute changes in the lower (min) or higher (max) detection recognition threshold. To evaluate whether these asymmetries affected vestibular function during natural bilateral activation, we compared direction recognition thresholds for real rotations and binaural virtual rotations between the AIS and control groups. For normally distributed data (evaluated using Shapiro-Wilk's tests), we used Student's *t*-tests (t_df_) to compare the direction recognition thresholds and Cohen's d to estimate effect size, whereas for non-normally distributed data, we used Mann-Whitney *U* tests (*U*) and Rank-Biserial Correlations (*r*), respectively. A Pearson's correlation (*r*^2^) was used to characterize the relationship between the recognition threshold asymmetry (mA) and the Cobb angle (°) of the scoliosis curvature. All statistical analyses were performed with JASP (v0.8.6, The JASP Team, The Netherlands). Results from parametric analyses were reported as means and standard deviations (SD) and those from non-parametric analyses as medians and the 1st and 3rd quartiles (42). Statistical significance was set at alpha (α) < 0.05.

## Results

**Figure 3:**
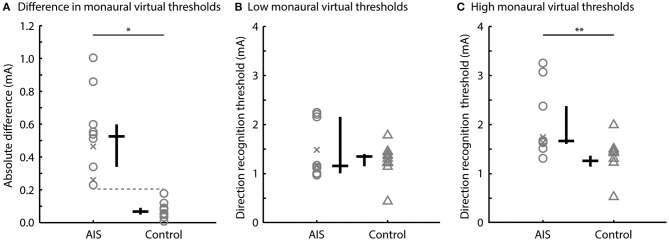
Direction recognition thresholds to monaural virtual rotations. Comparisons of **(A)** the absolute differences in left and right direction recognition thresholds, **(B)** the low direction recognition thresholds, and **(C)** the high direction recognition thresholds between the AIS and control subjects. Gray markers show individual data; black bars show the median and interquartile range. Post-surgery subjects are shown by the x markers (^*^p < 0.001; ^**^p < 0.01).

There was no overlap in the absolute difference in direction recognition threshold for the monaural configuration between participants with AIS (0.22–1.00 mA) and controls (0.01–0.21 mA) (*U* = 100.00, *p* < 0.001, *r* = 1.00; Figure 3A). AIS and control subjects exhibited similar low direction recognition thresholds [1.16 [1.04, 2.25] vs. 1.26 [1.15, 1.71] mA; *U* = 51.00, *p* = 0.97, *r* = 0.02] (Figure 3B), but AIS subjects exhibited larger high direction recognition thresholds compared to controls [1.67 [1.61, 3.25] vs. 1.35 [1.17, 1.91] mA; *U* = 88.00, *p* = 0.003; *r* = 0.76] (Figure 3C).

**Figure 4:**
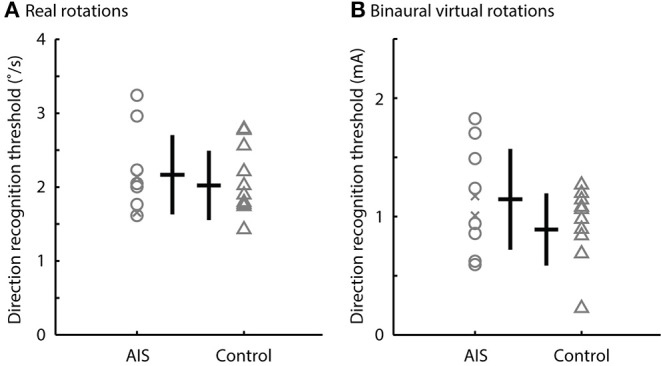
Direction recognition thresholds to real rotations and binaural virtual rotations. Comparisons of the direction recognition thresholds for **(A)** the real rotations and **(B)** the binaural virtual rotations generated by the EVS. There was no difference between the AIS subjects and controls (*p* > 0.05). Gray markers show individual data; black bars show the median and interquartile range. Post-surgery subjects are shown by the x markers.

There was no difference between the direction recognition thresholds of the real and bilateral virtual stimuli for the AIS and control subjects. The direction recognition thresholds for the real rotations ranged from 1.6 to 3.2°/s for participants with AIS and from 1.4 to 2.7°/s for control participants (t_18_ = 0.64, *p* = 0.53, *d* = 0.53) (Figure 4A). Similarly, the direction recognition thresholds for virtual rotations (binaural configuration) ranged from 0.59 to 1.82 mA for participants with AIS and from 0.18 to 1.15 mA for control participants (t_18_ = 1.54, *p* = 0.14, *d* = 0.69) (Figure 4B).

**Figure 5:**
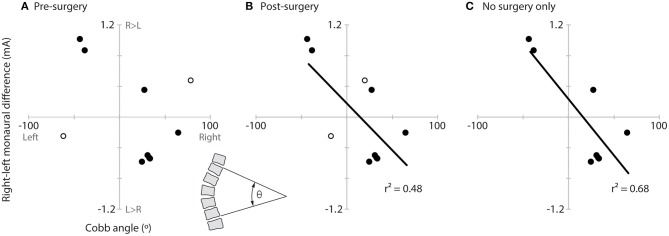
Relationship of vestibular asymmetry to Cobb angle. Plots of the vestibular asymmetry (right–left recognition threshold) vs. Cobb angle (left curve negative, right curve positive) and significant linear correlations (lines and *r*^2^) for **(A)** all AIS participants using the pre-surgery Cobb angle of the two post-surgery participants (hollow circles), **(B)** all AIS participants using the post-surgery Cobb angles of the two post-surgery participants (hollow circles), and **(C)** only the eight pre-surgery participants. The inset between panels **(A,B)** (bottom) illustrates how Cobb angle (θ) was measured.

No correlation was observed between the direction recognition threshold asymmetry and Cobb angle when using the pre-surgery Cobb angle for the two participants who were tested post-surgery (*r*^2^ = 0.14, *p* = 0.30; Figure 5A). Significant correlations were observed, however, when using these two subjects' post-surgery Cobb angle (*r*^2^ = 0.48, *p* = 0.03; Figure 5B) or when examining only the eight pre-surgery participants (*r*^2^ = 0.68, *p* = 0.01; Figure 5C).

## Discussion

The aim of this study was to investigate vestibular asymmetry in individuals with AIS and matched control participants. We estimated vestibular asymmetry by computing the absolute value of the difference between the left and right direction recognition thresholds using monaural electrical stimulation of the vestibular organs. Confirming our hypothesis, all individuals with AIS exhibited a larger functional vestibular asymmetry than age- and sex- matched control participants, i.e., a larger absolute difference between left and right direction recognition thresholds, than age- and sex- matched control participants. Further exploration of these data showed that the larger asymmetry in AIS participants was more likely the result of an abnormally high direction recognition threshold for one of their two vestibular organs; the direction recognition threshold of their other vestibular organs was not significantly different from controls.

### Asymmetric Vestibular Function in Participants With AIS

For virtual rotations elicited by monaural EVS, all participants with AIS exhibited a larger absolute difference between their left and right direction recognition thresholds than all of the matched control participants. This functional vestibular asymmetry in AIS participants appears to be related to an elevated direction recognitions threshold, i.e., poorer perception, for one of their two vestibular organs. Their other vestibular organ appears to have a direction recognition threshold that is similar to the control participants. The lack of overlap between the asymmetry seen in our AIS and control participants suggests that AIS is related to a unilateral vestibular deficit; however, numerous additional questions must be answered before a causal relationship can be inferred.

Using caloric vestibular stimulation, Sahlstrand et al. (6) reported that individuals with AIS exhibited an 8.6% asymmetry in their ocular responses compared to a 3.7% asymmetry observed in controls. Balance responses evoked by caloric vestibular stimuli were also more pronounced in individuals with AIS (7) although considerable overlap between their AIS and controls was present. Moreover, these asymmetric vestibular responses to caloric stimuli were not reproduced by Hitier et al. (9). Jensen and Wilson (43), on the other hand, compared the time duration of nystagmus following a rotary movement with the participant's head flexed 30 degrees to bias the horizontal semicircular canal. These authors reported a shorter nystagmus duration for AIS participants compared to controls regardless of direction of rotation (43). Our results support Sahlstrand's and Jensen et al.'s (7, 43) findings but indicate a larger effect size when estimating vestibular asymmetry with monaural EVS. The differences in vestibular asymmetry between studies could be related to preferential activation of the horizontal semicircular canals with caloric vestibular stimulation (11–13) compared to the activation of all vestibular afferents by EVS (17, 21, 24, 25). Another possible explanation for the clearer separation that we observed between AIS and control participants could be that we tested vestibular perception thresholds rather than ocular or balance function. While ocular and balance testing are more objective and presumably less variable than the subjective results of the perception threshold testing used here, the degree with which we were able to discriminate AIS and control participants using a subjective measure suggests that threshold behavior of the vestibular organs may more closely capture the underlying vestibular dysfunction present in AIS individuals.

One possible explanation for the asymmetry we observed is a difference in the orientation of the semi-circular canals of the AIS participants. Shi et al. (8) reported that the angle between the anterior and horizontal canals in AIS patients were up to 3 degrees different on the left side compared to the right side. Assuming that the net virtual motion induced by EVS results from a vector sum of all vestibular afferents in the semicircular canals and otoliths (17), a difference in canal orientation of this magnitude yields a 0.7-degree difference in the orientation of the net left and net right EVS vectors (for unilateral stimuli). When considering only the components of the left and right EVS vectors parallel to the Earth's vertical in a head down posture, this 0.7-degree angular difference would be the same as changing the EVS current by 0.007% between the left and right sides. For an average detection recognition threshold of 1.5 mA (Figures 3B,C), the expected difference in left- and right-side currents equates to about 0.0001 mA. This difference is significantly smaller than the difference between the left and right direction recognition thresholds we observed in participants with AIS (0.53 ± 0.27 mA; one-sample Wilcoxon signed-rank test, z = 2.80, one-tailed *p* = 0.0013). Based on this analysis, the asymmetric vestibular direction recognition thresholds we measured are not explained by anatomical asymmetries in semicircular canal orientation alone.

We did not observe a relationship between vestibular asymmetry and spine curvature when using the pre-surgery Cobb angles of the two participants we tested following surgery. This relationship was significant, however, when we used their post-surgery Cobb angles or when we removed these two surgical participants from the analysis entirely. The relationship showed that a higher right-side direction recognition threshold was related to a spine that curved to the left (and vice versa). Sahlstrand et al. (6) only correlated direction of curvature with direction of vestibular asymmetry, not the magnitude, but did not report a significant correlation. Our results of greater correlation of vestibular asymmetry to post-surgery Cobb angles seem to suggest that the vestibular asymmetry is influenced by the participant's curvature and could suggest a proprioceptive contribution to the vestibular asymmetry observed here. Asymmetric proprioceptive inputs from the trunk could influence central vestibular neurons and influence the perception of vestibular stimuli. More work is needed to further explore this possibility.

### Methodological Considerations

Although EVS activates all primary vestibular afferents (either directly or via the activation of hair cells), irregular afferents exhibit a larger sensitivity to the electrical stimuli (21, 23, 25). It may be tempting to connect the increased sensitivity to potential decreases in thresholds and suggest the thresholds observed here are mainly associated with the response of irregular afferents to electrical stimuli. However, Kwan et al. (21) showed that vestibular afferent thresholds to electrical vestibular stimulation do not differ across regular and irregular vestibular afferents. Instead, the increased variability of irregular afferents offsets any advantage provided by their increased sensitivity such that thresholds are similar between irregular and regular afferents. Consequently, we propose that the direction recognition thresholds reported in the present study could be explained by the activation of regular and irregular afferents to EVS.

In control participants, the direction recognition thresholds to virtual rotations elicited by binaural EVS were ~70% of those evoked by monaural EVS (0.89 mA vs. 1.24 mA). If monaural EVS was a purely unilateral vestibular stimulus, we would have expected this value to be ~50%. To replicate the observed direction recognition thresholds to virtual rotations elicited by binaural and monaural EVS, we had to consider that the additional 20% (i.e., 70–50%) was due to stimulation of the contralateral vestibular afferents during monaural stimulation. Assuming each vestibular organ contributes half of the net vestibular signal, this finding suggests that monaural stimulation causes the contralateral vestibular organ to generate 40% (i.e., 20%/50%) of the signal generated by the ipsilateral vestibular organ during monaural EVS. This prediction is in line with the non-linear vector summation of balance and ocular responses to EVS administered in monaural and binaural configurations (32, 38). As a result, monaural EVS cannot be interpreted as a pure unilateral activation of the vestibular system. Based on the orientation of the semi-circular canals reported by Della Santina (44), however, this 40% cross stimulation does not explain the vestibular functional asymmetry we observed. Accounting for the 40% cross stimulation, the asymmetric semicircular canal orientation reported by Shi et al. (8) explains <0.015% of the functional vestibular asymmetry observed here. Hence, we propose that the vestibular direction recognition asymmetry observed in individuals with AIS is related to a dysfunction of one vestibular apparatus and not related to either methodological considerations associated with EVS or semi-circular canal orientation differences.

### Vestibular Function to Bilateral Activation

The direction recognition thresholds of virtual rotations evoked by binaural EVS and real rotations were similar between participants with AIS and controls. This finding suggests that the vestibular asymmetry we observed to monaural vestibular stimuli seems to be, at least partly, compensated for during bilateral activation of the vestibular system. When processing vestibular information, the brain is used to integrating information from bilateral activation of the canals whereas unilateral activation of the semi-circular canals is uncommon. We propose that the integration of information from bilateral activation of the semi-circular canals is well (re)calibrated internally, leading to minor differences between AIS and controls in direction recognition thresholds of real rotations and virtual rotations evoked by binaural EVS. In non-human primates, direct recordings from vestibular afferents indicate that vestibular thresholds likely result from combining the activity of multiple neurons known to project to higher-order brain centers for conscious perception (45). This compensation may explain why prior researchers had difficulty identifying vestibular dysfunction in AIS using natural (and therefore bilateral) activation of vestibular afferents or EVS in a binaural configuration (46–48). In our dataset, only 3 of our 10 AIS participants exhibited larger vestibular direction recognition threshold to binaural EVS, suggesting that a larger sample size (*n* ≈ 20/group) would be needed to detect a significant difference between AIS participants and controls. Even with a larger sample, however, there would still remain a considerable overlap in the direction recognition thresholds of virtual rotations elicited by binaural EVS between AIS and control participants. These results suggest that direction recognition thresholds estimated using virtual rotations induced by monaural EVS have greater promise for yielding a diagnostic criterion with both a high sensitivity and specificity.

### Clinical Implications

Vestibular perception measurement is a simple, cost effective tool to assess vestibular function (34). Previous reports from caloric vestibular stimulation include participants who were not able to tolerate the stimulation due to vertigo and nausea (7). All of the participants in the present study enjoyed the experiment and tolerated the EVS well, with no one reporting vertigo or nausea. The methods and asymmetry metric used here could be an economical and novel way to assess/monitor scoliosis. The results from our study are very promising, but more research is needed to verify their utility as a prognostic tool or to develop a new modality to treat/manage scoliosis based on these findings.

### Limitations

The EVS methodology used here activates all primary vestibular afferents from the semicircular canals and otoliths with irregular afferents exhibiting a larger sensitivity to the electrical stimuli (21–23). Balance responses to monaural EVS suggest that there is little otolith contribution to the net movement vector but this phenomenon has not been fully assessed (26). Consequently, additional experiments specifically targeting otolithic function or the integration of information from the semi-circular canals and otoliths are required. Also, vestibular perception represents only one domain of vestibular function and other aspects of vestibular functions (ocular, balance, navigation) should be investigated. For example, Simoneau et al. (49) showed that participants with AIS exhibit impairments in their ability to memorize and process vestibular signals. To assess if participants with AIS who had undergone spinal fusion surgery influenced the data, we completed all of the above analyses with and without the individuals with AIS who received a surgery (*n* = 2) and their matched control participants (*n* = 2). No differences in the results were observed, and therefore we presented only the results from the combined AIS participants. However, the small number of participants with spinal fusion surgery does not allow us to draw any conclusions regarding the relative strength of the observed effects between AIS participants with and without spinal fusion surgery.

## Conclusion

Our results support the hypothesis that individuals with AIS have an asymmetric vestibular function that is clearly detectable with electrical vestibular stimuli applied in a monaural configuration. This functional vestibular asymmetry, however, is compensated during bilateral activation of the vestibular system. The methods used in this study can be readily adapted for assessing vestibular function in a clinical setting, ideally allowing for earlier intervention and mitigation of scoliotic curvature.

## Data Availability Statement

The data that support the findings of this study are available on request from the corresponding author. The data are not publicly available due to lack of prior ethical approval.

## Ethics Statement

The studies involving human participants were reviewed and approved by Clinical Research Ethics Board of the University of British Columbia. Written informed consent to participate in this study was provided by the participants' legal guardian/next of kin.

## Author Contributions

EW was the lead investigator on the project, responsible for concept formation, data collection and analysis, and document composition. J-SB was the supervisory author on the project and was involved in the concept formation, data analysis, and document composition. GS was involved in the concept development, data analysis, and document composition. CR was involved in the concept development and document composition.

### Conflict of Interest

GS is an employee, director and owns shares in MEA Forensic Engineers & Scientists, a forensic consulting company, and he may benefit from being involved in this study. The remaining authors declare that the research was conducted in the absence of any commercial or financial relationships that could be construed as a potential conflict of interest.
